# ERβ Accelerates Diabetic Wound Healing by Ameliorating Hyperglycemia-Induced Persistent Oxidative Stress

**DOI:** 10.3389/fendo.2019.00499

**Published:** 2019-07-24

**Authors:** Xueqing Zhou, Min Li, Meifang Xiao, Qiongfang Ruan, Zhigang Chu, Ziqing Ye, Liyan Zhong, Haimou Zhang, Xiaodong Huang, Weiguo Xie, Ling Li, Paul Yao

**Affiliations:** ^1^Department of General Surgery, Zhongnan Hospital of Wuhan University, Wuhan, China; ^2^Institute of Burns, Tongren Hospital of Wuhan University (Wuhan Third Hospital), Wuhan, China; ^3^Hainan Maternal and Child Health Hospital, Haikou, China; ^4^State Key Lab of Biocatalysis and Enzyme Engineering, School of Life Sciences, Hubei University, Wuhan, China

**Keywords:** ERβ, SOD2, mitochondria, oxidative stress, wound healing

## Abstract

Delayed wound healing in diabetic patients is a serious diabetic complication, resulting in major health problems as well as high mortality and disability. The detailed mechanism still needs to be fully understood. In this study, we aim to investigate potential mechanisms and explore an efficient strategy for clinical treatment of diabetic wound healing. Human umbilical endothelial cells were exposed to hyperglycemia for 4 days, then switched to normoglycemia for an additional 4 days. The cells were harvested for the analysis of reactive oxygen species (ROS) generation, gene expression and VEGF signaling pathway. Furthermore, the diabetic wound model was established in rats for the evaluation of wound healing rates under the treatment of either ERβ agonist/antagonist or SOD mimetic MnTBAP. Our results show that transient hyperglycemia exposure results in persistent ROS overgeneration after the switch to normoglycemia, along with suppressed expression of ERβ, SOD2, and the VEGF signaling pathway. Either ERβ expression or activation diminishes ROS generation. *In vivo* experiments with diabetic rats show that ERβ activation or SOD mimetic MnTBAP diminishes ROS generation in tissues and accelerates diabetic wound healing. Transient hyperglycemia exposure induces ROS generation and suppresses ERβ expression, subsequently resulting in SOD2 suppression with additional elevated ROS generation. This forms a positive-feed forward loop for ROS generation with persistent oxidative stress. ERβ expression or activation breaks this loop and ameliorates this effect, thereby accelerating diabetic wound healing. We conclude that ERβ accelerates diabetic wound healing by ameliorating hyperglycemia-induced persistent oxidative stress. This provides a new strategy for clinical treatment of diabetic wound healing based on ERβ activation.

## Introduction

Wound healing is involved with many tissues and factors, including endothelial cells, fibroblasts, and blood cells, together with many sequential process of inflammation, granulation formation and tissue remodeling. Diabetes delays wound healing due to its contribution to the defective regulation of complicated molecular and cellular events in the proper healing process (1). Diabetic foot ulcer (DFU) is one of the most serious diabetic complications that results from poor wound healing, which subsequently results in a major health problem in patients, causing high mortality and disability (2, 3). Diabetic wound healing is involved with multiple complex pathophysiological mechanisms with many extrinsic and intrinsic factors (4). Full understand of the detailed mechanism is still quite necessary for the development of an efficient clinical treatment strategy for diabetic wound healing (5, 6).

Hyperglycemia-induced over-generation of reactive oxygen species (ROS) and subsequent oxidative stress is a major contributor to diabetic complications, as this ROS initiates many pathological signaling pathways, resulting in diabetic tissue damage (7–9). The antioxidant enzyme SOD2 (mitochondrial superoxide dismutase) diminishes mitochondrial ${\text{O}}_{{2{}^{\cdot }}^{-}}$ and plays a protective role in this process. SOD2 suppression results in increased ROS generation with subsequent mitochondrial dysfunction and oxidative stress (10, 11); this may be one of the reasons for delayed wound healing in diabetic patients (12, 13).

Estrogen receptor β (ERβ) modulates the basal expression of SOD2, regulates oxidative stress (12) and mitochondrial function (14, 15), and plays a protective role in tissue damage. ERβ suppression results in oxidative stress and dysfunction of mitochondrial and lipid metabolism, subsequently triggering tissue damage with many pathological consequences and clinical symptoms (16). It has been reported that estrogen promotes wound healing by ERβ independent of its anti-inflammatory activities, while the related mechanism remains unclear (17).

In an effort to investigate the potential mechanism for delayed wound healing in diabetes, we explored the role of oxidative stress and ERβ during hyperglycemia in endothelial cells (18). We found that transient hyperglycemia exposure induces persistent oxidative stress with maintained suppression of ERβ, SOD2, and the VEGF (vascular endothelial growth factor) signaling pathway after switching to normoglycemia. Further investigation showed that hyperglycemia-induced oxidative stress down-regulates ERβ expression, and subsequently suppresses SOD2 expression with a positive-feed forward loop to maintain elevated ROS generation even in subsequent normoglycemia. Either ERβ expression or ERβ agonist DPN breaks this loop for ROS generation and diminishes hyperglycemia-induced oxidative stress. The further *in vivo* diabetic rat model showed that ERβ agonist DPN or SOD mimetic MnTBAP (19, 20) accelerates streptozotocin (STZ)-induced delayed diabetic wound healing, while ERβ antagonist PHTPP mimicked the diabetic effect in non-diabetic rats. We conclude that ERβ accelerates diabetic wound healing by ameliorating hyperglycemia-induced persistent oxidative stress.

## Materials and Methods

### Materials and Reagents

Primary Human Umbilical Vein Endothelial Cells (HUVECs, # CC-2935, obtained Lonza) were maintained in EGMTM-Plus Media (from Lonza) with all the supplements in addition to charcoal-stripped Fetal Bovine Serum (#12676029, Life Technologies) to remove traces of interfering basal estrogen. In some experiments, the HUVECs were conditionally immortalized by hTERT lentivirus vector with an extended life span to achieve higher transfection efficiency and experimental stability (21, 22). All cells were maintained in a humidified incubator with 5% CO_2_ at 37°C. The Hypoxia condition was induced by incubating in 94% N2, 5% CO2 and 1% O_2_ for 24 h.

Antibodies for β-actin (sc-47778), ERβ (sc-137381), HIF1α (sc-13515), SOD2 (sc-30080), and VEGF (sc-7269) were obtained from Santa Cruz Biotechnology. The antibody for CD31 (ab24590) was obtained from Abcam. 3-nitrotyrosine (3-NT) was measured by 3-Nitrotyrosine ELISA Kit (ab116691 from Abcam), and the HIF1α transcriptional activity was measured by HIF1α Transcription Factor Assay Kit (ab113104 from Abcam) in 50 μl nuclear extracts from treated cells per manufacturers' instructions. Nuclear extracts were prepared using the NE-PER Nuclear and Cytoplasmic Extraction Reagents Kit (Pierce Biotechnology). The mitochondrial fraction was isolated using a Pierce Mitochondria Isolation Kit (Pierce Biotechnology) according to manufacturers' instructions. The protein concentration was measured using the Coomassie Protein Assay Kit (Pierce Biotechnology) per manufacturers' instructions. Luciferase activity assay was carried out using the Dual-Luciferase™ Assay System (Promega) and the transfection efficiency was normalized using a cotransfected renilla plasmid (23).

ERα agonist PPT (#1426), ERβ agonist DPN (#1494) and ERβ antagonist PHTPP (#2662) were obtained from Tocris. Streptozotocin (STZ, #18883-66-4), and MnTBAP, a cell-permeable superoxide dismutase (SOD) mimetic and peroxynitrite scavenger (#475870), were obtained from Sigma.

### Construction of SOD2/VEGF Reporter Plasmid

The human genomic DNA was prepared from HUVECs cells. In order to construct SOD2/VEGF reporter plasmids, the SOD2 gene promoter (Ensembl gene ID: SOD2 ENST00000337404) and VEGF gene promoter (Ensembl gene ID: VEGFA-201 ENST00000230480.10) were amplified by PCR and subcloned into the pGL3-basic vector (# E1751, Promega) using restriction sites of Mlu I and Hind III with the following primers: SOD2 forward: 5′-gcgc-acgcgt- gaa tcc tgt gga ttc atc ctt−3′ (Mlu I) and SOD2 reverse: 5′- gtac- aagctt- ctg aag acg aga aag cac agc−3′ (Hind III); VEGF forward: 5′-gcgc-acgcgt- ctg tga acc ttg gtg ggg gtc−3′ (Mlu I) and VEGF reverse: 5′- gtac- aagctt -ctc gag agg tca cct tcc cgc−3′ (Hind III). All the vectors were verified by sequencing, and detailed information on these plasmids is available upon request (23).

### Generation of Human ERβ/SOD2 Expression Lentivirus

The human cDNA for ERβ and SOD2 was obtained from Open Biosystems. The cDNA for either human ERβ or SOD2 was subcloned into the pLVX-Puro vector (from Clontech) with the restriction sites of Xho1 and Xba1 using the below primers: ERβ forward primer: 5′- gtac - ctcgag- atg gat ata aaa aac tca cca−3′ (Xho1) and ERβ reverse primer: 5′- gtac - tctaga- tca ctg ctc cat cgt tgc ttc−3′ (Xba1); SOD2 forward primer: 5′- gtac- ctcgag- atg ttg agc cgg gca gtg tgc−3′ (Xho1) and SOD2 reverse primer: 5′- gtac- tctaga- tta ctt ttt gca agc cat gta−3′ (Xba1). The ERβ/SOD2 or empty control (CTL) was expressed through Lenti-X™ Lentiviral Expression Systems (from Clontech) per manufacturers' instructions (16).

### Measurement of ROS Generation

Treated cells were seeded in a 24-well plate and incubated with 10 μM CM-H2DCFDA (Invitrogen) for 45 min at 37°C, and then the intracellular formation of reactive oxygen species (ROS) was measured at excitation/emission wavelengths of 485/530 nm using a FLx800 microplate fluorescence reader (Bio-Tek). The data was normalized as arbitrary units (23, 24).

### RT Reaction and Real-Time Quantitative PCR

Total RNA from treated cells was extracted using the RNeasy Micro Kit (Qiagen), and the RNA was reverse transcribed using an Omniscript RT kit (Qiagen). All the primers were designed using Primer 3 Plus software with the Tm at 60°C, primer size of 21bp, and the product length in the range of 140–160 bp (see Table S1). The primers were validated with the amplification efficiency in the range of 1.9–2.1, and the amplified products were confirmed with agarose gel. The real-time quantitative PCR was run on iCycler iQ (Bio-Rad) with the Quantitect SYBR green PCR kit (Qiagen). The PCR was performed by denaturing at 95°C for 8 min, followed by 45 cycles of denaturation at 95°C, annealing at 60°C, and extension at 72°C for 10 s, respectively. One microliter of each cDNA was used to measure target genes. The β-actin was used as the housekeeping gene for transcript normalization, and the mean values were used to calculate relative transcript levels with the ^ΔΔ^CT method per instructions from Qiagen. In brief, the amplified transcripts were quantified by the comparative threshold cycle method using β-actin as a normalizer. Fold changes in gene mRNA expression were calculated as 2^−Δ*ΔCT*^ with CT = threshold cycle, ΔCT = CT (target gene)-CT(β-actin), and the ΔΔCT = ΔCT (experimental)-ΔCT (reference) (16, 23).

### Western Blotting

Cells were lysed in an ice-cold lysis buffer (0.137M NaCl, 2mM EDTA, 10% glycerol, 1% NP-40, 20 mM Tris base, pH 8.0) with protease inhibitor cocktail (Sigma). The proteins were separated in 10% SDS-PAGE and further transferred to the PVDF membrane. The membrane was incubated with appropriate antibodies, washed and incubated with HRP-labeled secondary antibodies, and then the blots were visualized using the ECL+plus Western Blotting Detection System (Amersham). The blots were quantitated by IMAGEQUANT, and final results were normalized by β-actin (16, 23).

### Luciferase Reporter Assay

1.0 ×10^5^ of SNK-6 cells were seeded in a 6-well plate with complete medium to grow until they reached 80% confluence. Cells were then cotransfected by 3 μg of VEGF full length or deletion reporter constructs, together with 0.2 μg of pRL-CMV-Luc *Renilla* plasmid (from Promega). Then, cells were treated by either 5 mM aspirin or empty control (CTL) for 24 h. After treatment, the cells were harvested and the luciferase activity assays were carried out using the Dual-Luciferase™ Assay System (Promega), and the transfection efficiencies were normalized using a cotransfected *Renilla* plasmid according to manufacturers' instructions. The VEGF reporter activity from different treatments was calculated (23).

### Chromatin Immunoprecipitation (ChIP)

Cells were washed and crosslinked using 1% formaldehyde for 20 min and terminated by 0.1M glycine. Cell lysates were sonicated and centrifuged. Five hundred microgram of protein were pre-cleared by BSA/salmon sperm DNA with preimmune IgG and a slurry of Protein A Agarose beads. Immunoprecipitations were performed with the indicated antibodies, BSA/salmon sperm DNA and a 50% slurry of Protein A agarose beads. Input and immunoprecipitates were washed and eluted, then incubated with 0.2 mg/ml Proteinase K for 2 h at 42°C, followed by 6 h at 65°C to reverse the formaldehyde crosslinking. DNA fragments were recovered by phenol/chloroform extraction and ethanol precipitation. A ~150 bp fragment on the SOD2 or VEGF promoter was amplified by real-time PCR (qPCR) using the primers provided in Table S1 (16, 23).

### SOD2 Activity Assay

The SOD2 was obtained from the mitochondrial fraction that was isolated using a Pierce Mitochondria Isolation Kit (Pierce) according to manufacturers' instructions. SOD activity was measured as described previously (25). In brief, a stable O2^.−^ source was generated through the conversion action of XOD (xanthine oxidase) from xanthine and was mixed with chemiluminescent (CL) reagents to achieve a stable light emission. The SOD2 sample injection can scavenge ${\text{O}}_{2}^{.-}$ and the subsequent decrease of chemiluminescent response is proportional to the SOD2 activity. This system can have a detection limit of 0.001 U.ml-1 with the linear range of 0.03~2.00 U.ml-1. The results were normalized by protein concentration and expressed as Units/mg proteins (U/mg) (15).

### *In vivo* Rat Experiments

The animal protocol conformed to US NIH guidelines (Guide for the Care and Use of Laboratory Animals, No. 85–23, revised 1996), and was reviewed and approved by the Institutional Animal Care and Use Committee from Wuhan University. The female rats were housed 4 or 5 per cage on a 12:12-h light-dark cycle and were given phytoestrogen-free commercial rodent chow and water *ad libitum* on arrival. Estrous cycles were monitored with daily vaginal smears. Only rats with at least two regular 4 to 5 d estrous cycles were included in the studies. 6-week old females were anesthetized using a ketamine/xylazine mixture (80 and 4 mg/kg, respectively, intramuscular), and ovariectomized (OVX), then the OVX rats were ready for further experiments after 1-week recovery (26).

#### Diabetic Rat Model

Chronic diabetic rats were induced by injection of 50 mg/kg streptozotocin (STZ, 0.05 M sodium citrate, pH 5.5) after an 8 h fasting. Animals with blood glucose >300 mg/dl were considered positive, while control (CTL) rats received only vehicle injection.

#### Rat Models of Cutaneous Burn

Wild type and diabetic rats were subjected to a model of cutaneous burn injury. The dorsum of each rat was shaved with electric clippers and depilated with Nair. The rats were anesthetized by inhalation of 5% isoflurane, and then the cutaneous burn injury was made on the dorsa of the rats by exposure to a hot copper pillar (2-cm diameter) at 75°C for 15 s.

#### Experimental Groups

The experimental rats were separated into 5 groups: Group 1. Wild type (CTL) rats received only subcutaneously vehicle (5% DMSO in maize oil) injection; Group 2. Diabetic (STZ) rats received only vehicle injection; Group 3. Diabetic (STZ) rats received 450 μg/kg/day of DPN (dissolved in DMSO) injection on days of 0, 4, 8, 12, and 16 with respect to the time of wounding (STZ/DPN); Group 4. STZ rats received 10 mg/kg/day of MnTBAP (dissolved in DMSO) injection (STZ/MnTBAP); Group 5. CTL rats received 450 μg/kg/day of PHTPP (dissolved in DMSO) injection (CTL/PHTPP).

#### Measurement

Digital photographs of the wounds were taken every 2 days for 20 days. Wound area was quantified as a percent area of the original wound size using Image J software. At indicated time points, wounds were excised and snap-frozen or, alternatively, processed for H&E staining. Vascular density was detected on frozen sections by immunohistochemistry using CD31 mouse monoclonal antibody. For quantification of CD31 positivity, wounds were analyzed under 200^*x*^ magnification, and the number of positive 6 cells per high-power field (HPF) were counted. All counts and observations were performed by a blinded observer (27).

### Immunohistochemistry

The tissues were dissected and snap-frozen in the OCT compound. The 10 μm sections were cut by clean microtome and mounted on PEN-membrane slides (2.0 μm, Leica), and stored in −20°C before use. The slides were first fixed by 3.7% formaldehyde at 37°C for 15 min, then permeabilized by 1% BSA+0.2% Triton X-100 in PBS for 1 h, then blotted with 40 μg/ml (dilute 1:20) of either VEGF or CD31 mouse monoclonal antibody for 2 h, washed three times and the Texas-red labeled anti-mouse secondary antibody (1:200) was added for blotting for another 1 h. After thorough washing, the slides were visualized and photographed. The relative densities of each group were quantitated for protein expression using Image J. software (22).

### *In vivo* Superoxide Release

Superoxide anion (${\text{O}}_{{2{}^{\cdot }}^{-}}$) release from the tissue was determined by a luminol-EDTA-Fe enhanced chemiluminescence (CL) system supplemented with DMSO-TBAC (Dimethyl sulfoxide-tetrabutyl-ammonium chloride) solution for extraction of released ${\text{O}}_{{2{}^{\cdot }}^{-}}$ from tissues as described previously (24). The superoxide levels were calculated from the standard curve generated by the xanthine/xanthine oxidase reaction (23).

### Statistical Analysis

The data was given as mean ± SEM; all of the experiments were performed at least in quadruplicate unless otherwise indicated. The one-way ANOVA followed by the Bonferroni *post hoc* test was used to determine statistical significance of different groups. The mouse survival curve was determined by Kaplan-Meier survival analysis using SPSS 22 software and a *P*-value < 0.05 was considered significant (23).

## Results

### ERβ Expression Diminishes Hyperglycemia-Induced Persistent ROS Generation

**Figure 1:**
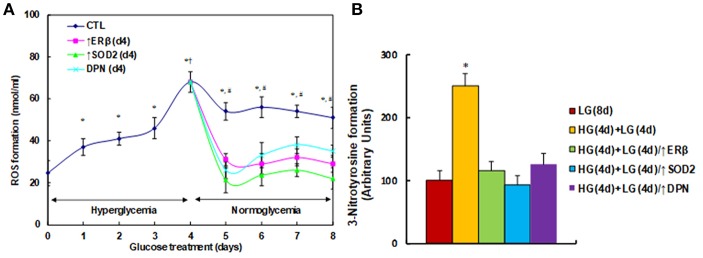
ERβ expression diminishes hyperglycemia-induced persistent ROS generation. **(A)** HUVECs were incubated in hyperglycemia (25 mM glucose) for 4 days, and then switched to normoglycemia (5 mM glucose) for an additional 4 days. On day 4, cells were infected by an empty vector (CTL), ERβ lentivirus (ERβ↑), or SOD2 lentivirus (SOD2↑), or treated by 100μM ERβ agonist (DPN) for a subsequent 4 days. The cells were harvested each day for analysis of ROS formation, *n* = 4. *P < 0.05, vs. day 0 group; †*P* < 0.05, vs. day 3 group; ^#^*P* < 0.05, vs. day 4 group. **(B)** The HUVECs cells were treated either in normoglycemia (5mM) for 8 days (LG(8d)), or in hyperglycemia (HG in 25mM glucose) for 4 days followed by normoglycemia (LG in 5mM glucose) for an additional 4 days (HG(4d)+LG(4d)), or the cells were infected at day 4 by either ERβ lentivirus (HG(4d)+LG(4d)/ERβ↑), or SOD2 lentivirus (HG(4d)+LG(4d)/SOD2↑), or treated by ERβ agonist (HG(4d)+LG(4d)/DPN) for subsequent 4 days, and the cells were harvested for the analysis of 3-nitrotyrosine formation, *n* = 5. *P < 0.05, vs. LG(8d) group. Data are expressed as mean ± SEM.

We first measured the effect of hyperglycemia on ROS generation in the HUVECs. The HUVECs were firstly exposed to high glucose (25 mM) for 4 days, then switched to low glucose (5 mM) for additional 4 days. We found that ROS generation slightly increased, continuously increasing to 187% on day 3, and then significantly increasing to 276% on day 4 compared to day 0. On day 5, the ROS generation slightly decreased, but maintained high levels with a 220% increase. These high levels of ROS lasted for 4 days with a 207% increase (on day 8) compared to day 0. On the other hand, the elevated ROS generation completely diminished after infection of either ERβ lentivirus (↑ERβ), or SOD2 lentivirus (↑SOD2), or treated by 100μM ERβ agonist (DPN) on day 4 (see Figure 1A). We also evaluated the potential effect of ERα agonist PPT, and found that 100μM PPT treatment on day 4 (PPT(d4)) had no effect on ROS generation (see Figure S1). We then measured the 3-nitrotyrosine (3-NT) generation. The results showed that 3-NT generation remains high after 4-day hyperglycemia treatment followed by 4-day normoglycemia (HG(4d)+LG(4d)) compared to the 8-day normoglycemia treatment (LG(8d)) group. Again, infection of either ERβ lentivirus (HG(4d)+LG(4d)/↑ERβ), or SOD2 lentivirus (HG(4d)+LG(4d)/↑SOD2), or treatment with 100μM ERβ agonist (HG(4d)+LG(4d)/↑DPN) on day 4 completely restored 3-NT generation to normal (see Figure 1B). Our results indicate that expression of either ERβ or SOD2, or ERβ agonist treatment, diminishes hyperglycemia-induced persistent ROS generation.

### ERβ Expression Diminishes Hyperglycemia-Induced Persistent SOD2 Suppression

**Figure 2:**
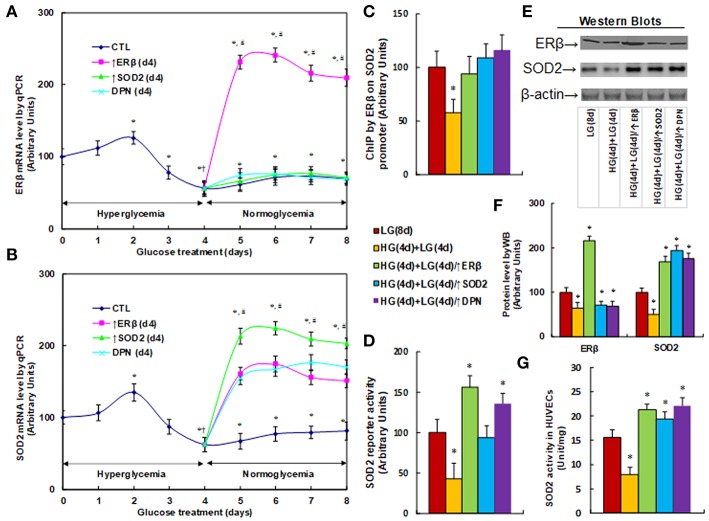
ERβ expression diminishes hyperglycemia-induced persistent SOD2 suppression. **(A,B)** HUVECs were incubated in hyperglycemia (25 mM glucose) for 4 days, and then switched to normoglycemia (5 mM glucose) for an additional 4 days. On day 4, cells were either infected by empty vector (CTL), ERβ lentivirus (ERβ↑), or SOD2 lentivirus (SOD2↑), or treated by 100 μM ERβ agonist (DPN) for a subsequent 4 days, and the cells were harvested for mRNA analysis of ERβ **(A)** and SOD2 **(B)**, *n* = 4. *P < 0.05, vs. day 0 group; †*P* < 0.05, vs. day 3 group; ^#^*P* < 0.05, vs. day 4 group. **(C-G)** The HUVECs cells were treated either in normoglycemia (5 mM) for 8 days (LG(8d)), or in hyperglycemia (HG in 25 mM glucose) for 4 days followed by normoglycemia (LG in 5 mM glucose) for an additional 4 days (HG(4d)+LG(4d)), or the cells were infected at day 4 by either ERβ lentivirus (HG(4d)+LG(4d)/ERβ↑), or SOD2 lentivirus (HG(4d)+LG(4d)/SOD2↑), or treated by ERβ agonist (HG(4d)+LG(4d)/DPN) for a subsequent 4 days, and the cells were harvested for further analysis. **(C)** ChIP analysis by ERβ antibody on SOD2 promoter, *n* = 4. **(D)** SOD2 reporter activity assay, *n* = 5. **(E)** Representative picture for western blots. **(F)** Protein quantitation for **(E)**, *n* = 4. **(G)** SOD2 activity assay, *n* = 5. *P < 0.05, vs. LG(8d) group. Data are expressed as mean ± SEM.

We measured the effect of hyperglycemia on gene expression in HUVECs. The HUVECs were firstly exposed to high glucose (25 mM) for 4 days, then switched to low glucose (5mM) for an additional 4 days as shown in Figure 1. We then measured the mRNA expression of ERβ (see Figure 2A) and SOD2 (see Figure 2B). The results showed that ERβ expression slightly increased to 126% on day 2, and then started to decrease on day 3. It decreased to 56% on day 4, and after switching from hyperglycemia to normoglycemia on days 5, 6, 7 and 8, the expression of both ERβ and SOD2 remained as low as on day 4. Infection of ERβ lentivirus (↑ERβ) on day 4 significantly increased expression of ERβ and SOD2, and the expression remained high throughout the next 4 days. On the other hand, either infection of SOD2 lentivirus (↑SOD2), or ERβ agonist (DPN) treatment significantly increased SOD2 expression, but did not increase ERβ expression. We also evaluated the potential effect of ERα agonist PPT, and found that 100μM PPT treatment on day 4 (PPT(d4)) had no effect on the expression of either ERβ or SOD2 (see Figure S2). These results indicate that ERβ may be the upstream target gene of SOD2. We then measured the binding ability of ERβ on the SOD2 promoter by ChIP analysis (see Figure 2C). It showed that ERβ binding ability decreased to 58% as a result of HG(4d)+LG(4d) treatment compared to the LG(8d)) group, and this was completely restored by either infection of ERβ lentivirus (↑ERβ) or SOD2 lentivirus (↑SOD2), or ERβ agonist (DPN) treatment. We also measured SOD2 luciferase reporter activity (see Figure 2D). We found that ERβ binding ability decreased to 58% as a result of HG(4d)+LG(4d) treatment compared to LG(8d) group, and this was completely restored by SOD2 lentivirus infection (↑SOD2). The infection of ERβ lentivirus (↑ERβ) or ERβ agonist (DPN) treatment further increased SOD2 reporter activity to 156 and 135%, respectively. We also measured the protein expression for ERβ and SOD2 (see Figures 2E,F). The results showed that ERβ protein decreased to 64% as a result of HG(4d)+LG(4d) treatment compared to the LG(8d) group, and infection of ERβ lentivirus (↑ERβ) increased ERβ expression to 216%, while either SOD2 lentivirus infection (↑SOD2), or ERβ agonist (DPN) treatment showed no effect. On the other hand, SOD2 protein decreased to 51% as a result of HG(4d)+LG(4d) treatment compared to the LG(8d) group, and the lentivirus infection of either ERβ (↑ERβ) or SOD2 (↑SOD2), or ERβ agonist (DPN) treatment, increased SOD2 protein level by 169, 194, and 176%, respectively. Finally, we measured the SOD2 enzyme activity (see Figure 2G). The results showed that the activity decreased to 51% as a result of HG(4d)+LG(4d) treatment compared to the LG(8d) group, and the lentivirus infection of either ERβ (↑ERβ), SOD2 (↑SOD2), or ERβ agonist (DPN) treatment increased SOD2 enzyme activity by 137, 124, and 142%, respectively. Our results indicate that SOD2 expression is regulated by ERβ, and ERβ overexpression by lentivirus or ERβ activation by ERβ agonist DPN upregulates SOD2 expression and subsequently diminishes hyperglycemia-induced persistent ROS generation.

### ERβ Expression Diminishes Hyperglycemia-Induced Persistent Suppression of the HIF1α/VEGF Signaling Pathway

**Figure 3:**
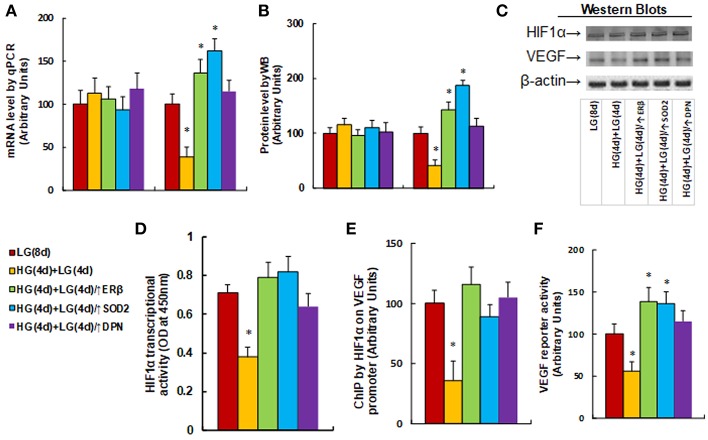
ERβ expression diminishes hyperglycemia-induced persistent suppression of the HIF1α/VEGF signaling pathway. The HUVECs cells were cultured in hypoxia conditions and treated either in normoglycemia (5 mM) for 8 days (LG(8d)), or in hyperglycemia (HG in 25 mM glucose) for 4 days followed by normoglycemia (LG in 5 mM glucose) for an additional 4 days (HG(4d)+LG(4d)), or the cells were infected at day 4 by either ERβ lentivirus (HG(4d)+LG(4d)/ERβ↑), or SOD2 lentivirus (HG(4d)+LG(4d)/SOD2↑), or treated by ERβ agonist (HG(4d)+LG(4d)/DPN) for subsequent 4 days, and the cells were harvested for further analysis. **(A)** mRNA levels by qPCR, *n* = 4. **(B)** Protein quantitation for western blots, *n* = 4. **(C)** Representative pictures for **(B)**. **(D)** HIF1α transcriptional activity assay, *n* = 5. **(E)** ChIP analysis by HIF1α antibody on VEGF promoter, *n* = 4. **(F)** VEGF reporter activity assay, *n* = 5. *P < 0.05, vs. LG(8d) group. Data are expressed as mean ± SEM.

We measured the effect of hyperglycemia on the HIF1α/VEGF signaling pathway. The HUVECs were cultured in hypoxia conditions and were treated first in high glucose (25 mM) for 4 days, and then switched to low glucose (5 mM) for an additional 4 days as described in Figure 1B. We first measured the mRNA expression levels for HIF1α and VEGF (see Figure 3A). The results showed that HIF1α mRNA levels have no significant changes in different treatments, while the VEGF mRNA levels decreased to 39% as a result of HG(4d)+LG(4d) treatment, and the infection of either ERβ lentivirus (↑ERβ) or SOD2 lentivirus (↑SOD2), or ERβ agonist DPN increased VEGF mRNA to 136, 162, and 115%, respectively compared to the LG(8d) group. We then measured protein expression through western blotting (see Figures 3B,C). The results showed that HIF1α protein levels have no significant changes under different treatments, while the VEGF protein level decreased to 41% as a result of HG(4d)+LG(4d) treatment, and the infection of either ERβ lentivirus (↑ERβ) or SOD2 lentivirus (↑SOD2), or treatment of ERβ agonist DPN, increased VEGF protein to 143, 187, and 113%, respectively compared to the LG(8d) group. We then measured the HIF1α transcriptional activity (see Figure 3D). The results showed that HIF1α transcriptional activity decreased to 53% as a result of HG(4d)+LG(4d) treatment compared to the LG(8d) group, and the infection of either ERβ lentivirus (↑ERβ) or SOD2 lentivirus (↑SOD2), or ERβ agonist DPN treatment, completely restored this effect. We also measured the HIF1α binding ability on the VEGF promoter by ChIP analysis (see Figure 3E). It showed that HIF1β binding ability was decreased to 36% by HG(4d)+LG(4d) treatment compared to LG(8d)) group, and this was completely restored by either infection of ERβ lentivirus (↑ERβ) or SOD2 lentivirus (↑SOD2), or ERβ agonist (DPN) treatment. Finally, we measured the VEGF luciferase reporter activity (see Figure 3F). The results showed that VEGF reporter activity decreased to 56% as a result of HG(4d)+LG(4d) treatment, and the infection of either ERβ lentivirus (↑ERβ) or SOD2 lentivirus (↑SOD2), or ERβ agonist DPN, increased VEGF reporter activity to 139, 136, and 115%, respectively compared to the LG(8d) group. The results indicated that VEGF expression is regulated by HIF1α under hypoxia conditions, and the expression of either ERβ or SOD2, or ERβ agonist DPN treatment, diminishes hyperglycemia-induced persistent ROS generation, activates HIF1α transcriptional activity, and subsequently upregulates VEGF expression, favoring wound healing.

### ERβ Activation Restores Diabetes-Induced Persistent Oxidative Stress and VEGF Suppression

**Figure 4:**
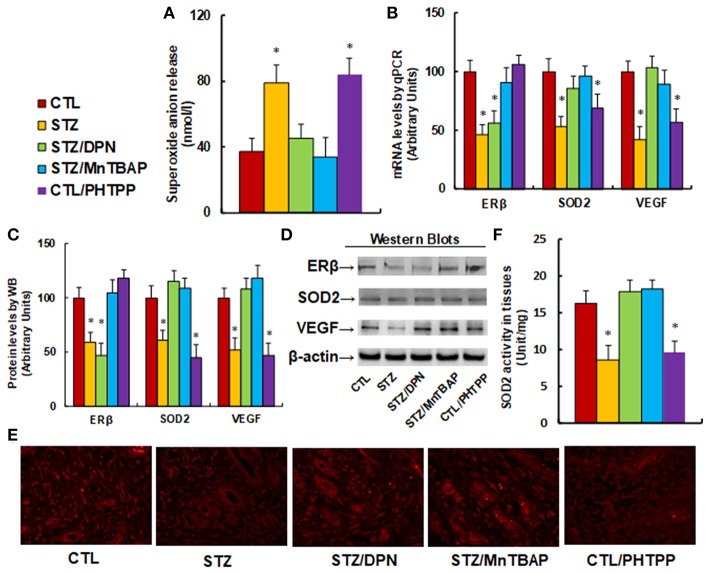
ERβ activation restores diabetes-induced persistent oxidative stress and VEGF suppression. The wounds were excised and collected from the experimental rats with either control rats (CTL), or STZ diabetic rats (STZ), or STZ rats treated by 450 μg/kg/day of DPN (STZ/DPN), or STZ rats treated by 10 mg/kg/day of MnTBAP (STZ/MnTBAP), or CTL rats treated by 450 μg/kg/day of PHTPP (CTL/PHTPP) for further analysis. **(A)** Superoxide anion release, *n* = 5. **(B)** mRNA levels by qPCR, *n* = 4. **(C)** Protein quantitation for western blots, *n* = 4. **(D)** Representative pictures for **(C)**. **(E)** Representative pictures for VEGF expression by immunohistochemistry. **(F)** SOD2 activity assay, *n* = 5. *P < 0.05, vs. CTL group. Data are expressed as mean ± SEM.

We evaluate the potential effect of ERβ activation on oxidative stress and the VEGF signaling pathway in diabetic wound healing in rats. The burn injury was introduced in either control (CTL) or diabetic (STZ) rats, and then treated with either ERβ agonist DPN (STZ/DPN), or SOD mimetic MnTBAP (STZ/MnTBAP), or ERβ antagonist PHTPP (CTL/PHTPP), and the wound tissues were collected for the further analysis. We first measured the superoxide anion release from the wound tissues (see Figure 4A). The results showed that superoxide anion (${\text{O}}_{{2{}^{\cdot }}^{-}}$) release increased to 214% in the STZ group compared to the CTL group. Either DPN (STZ/DPN) or MnTBAP treatment (STZ/MnTBAP) in STZ rats completely restored this effect, while PHTPP treatment in CTL rats (CTL/PHTPP) increased superoxide anion release to 227% compared to the CTL group, mimicking the effect of STZ rats. We also measured the mRNA expression of ERβ, SOD2 and VEGF (see Figure 4B). The results showed that ERβ expression was decreased to 46% in the STZ group compared to the CTL group, and both ERβ agonist DPN and antagonist PHTPP showed no effect on ERβ expression, while SOD mimetic MnTBAP (STZ/MnTBAP) completely restored the effect of STZ. In addition, the mRNA expression of SOD2 and VEGF decreased to 53 and 42%, respectively, in STZ treatment, and this effect was completely restored by DPN (STZ/DPN) and MnTBAP (STZ/MnTBAP) treatment. On the other hand, the mRNA expression of SOD2 and VEGF decreased to 69 and 57%, respectively, in the PHTPP treatment (CTL/PHTPP), mimicking the effect of STZ treatment. We then measured the protein expression, and a pattern similar to that of mRNA expression was observed for the protein levels of ERβ, SOD2 and VEGF (see Figures 4C,D). We also evaluated VEGF expression using immunohistochemistry (see Figure 4E), and the results showed that the VEGF staining in wound tissues was consistent with the results of the western blots as shown in Figure 4C. Finally, we measured the SOD2 enzyme activity in wound tissues (see Figure 4F). The results showed that SOD2 activity was decreased to 53% in STZ group compared to CTL group, and this effect was completely restored by DPN (STZ/DPN) and MnTBAP (STZ/MnTBAP) treatment, and the PHTPP treatment (CTL/PHTPP) mimicked the effect of STZ group. The results indicate that ERβ activation restores diabetes-induced persistent oxidative stress and VEGF suppression.

### ERβ Activation Accelerates Wound Healing in Diabetic Rats

**Figure 5:**
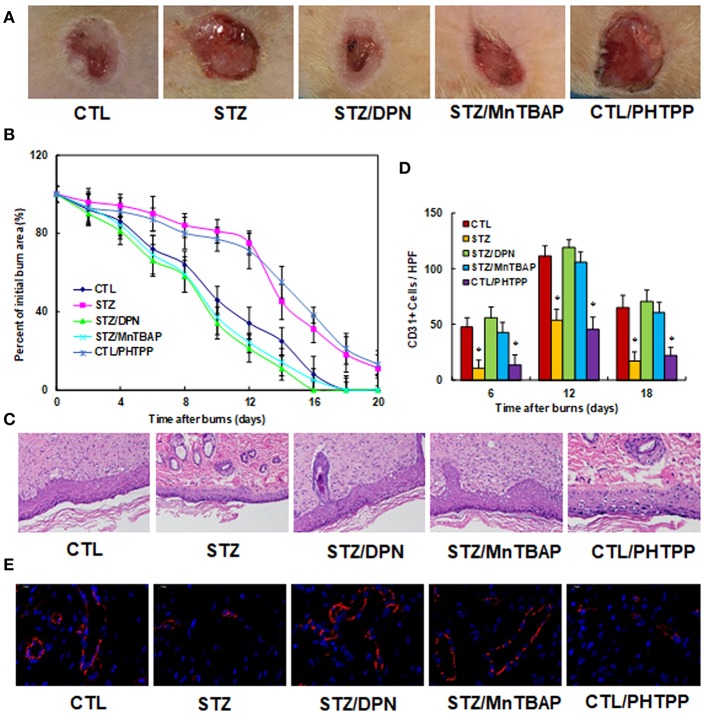
ERβ activation accelerates wound healing in diabetic rats. The experimental rats were divided into 5 groups: control (CTL), diabetic (STZ) treatment, STZ rats treated by 450 μg/kg/day of DPN (STZ/DPN), STZ rats treated by 10 mg/kg/day of MnTBAP (STZ/MnTBAP), or CTL rats treated by 450 μg/kg/day of PHTPP (CTL/PHTPP), for further analysis. **(A)** Photographs of representative wounds on day 12 after burns. **(B)** Graphical depiction of wound area in different days after burns. **(C)** H&E stains of wound tissue on day 12 after burns with occurrence of granulation tissue in the wounds. **(D)** Numbers of CD31 positive vessels per HPF area on day 12 after burns. **(E)** The representative pictures for the evaluation of vascularity (assessed by CD31 immunohistochemistry) on day 12 after burns. *N* = 7. *P < 0.05, vs. CTL group. Data are expressed as mean ± SEM.

We evaluated the potential effect of ERβ activation on wound healing in rats with burn injuries. The burn injury was introduced in either control (CTL) or diabetic (STZ) rats, and then treated with ERβ agonist DPN (STZ/DPN), SOD mimetic MnTBAP (STZ/MnTBAP), or ERβ antagonist PHTPP (CTL/PHTPP), and rate of wound healing was evaluated. We first evaluated the wound healing rate in different treatments. In Figure 5A, the representative pictures for wound area were taken on day 12, and the results showed that STZ rats had significantly delayed wound healing compared to CTL group, and the treatments of DPN (STZ/DPN) and MnTBAP (STZ/MnTBAP) completely restored this effect, while the PHTPP treatment in control rats (CTL/PHTPP) mimicked the effect of STZ rats. In Figure 5B, the relative wound areas were quantitated following different time points, and the results showed that STZ rats and CTL/PHTPP group had significantly delayed wound healing compared to groups of CTL, CTL/DPN and CTL/MnTBAP. We then evaluated the granulation tissue deposition by H&E staining on day 12 after the introduction of the burn injury (see Figure 5C). The results showed that the STZ group had significant less granulation tissue deposition compared to the CTL group. Both DPN (STZ/DPN) and MnTBAP (STZ/MnTBAP) treatments in STZ rats restored this effect, while PHTPP in CTL rats (CTL/PHTPP) mimicked the effect of STZ rats. Finally, we measured neovascularization by evaluating CD31^+^ positive (CD31^+^) cells using CD31 immunohistochemistry staining (see Figures 5D,E). In Figure 5D, the CD31^+^ cells were decreased to 23, 48, and 26%, respectively on days 6, 12 and 18 in STZ rats compared to CTL rats; this effect was completely restored by treatments of DPN (STZ/DPN) and MnTBAP (STZ/MnTBAP), while the PHTPP treatment in CTL rats (CTL/PHTPP) mimicked the effect of STZ rats. In addition, the representative pictures of CD31 staining were taken for the wound tissues on day 12 (see Figure 5E), the results showed the consistency with the quantitative details as shown in Figure 5D. Our results indicate that ERβ activation accelerates wound healing in diabetic rats.

## Discussion

In this study, we demonstrated that transient hyperglycemia exposure induces maintained ROS generation, resulting in suppression of ERβ and SOD2 and forming a positive feed-forward loop for ROS generation in subsequent normoglycemia. ERβ expression breaks this loop and restores hyperglycemia-induced oxidative stress. Furthermore, *in vivo* diabetic rat models showed that ERβ agonist DPN treatment ameliorates hyperglycemia-induced ROS generation and accelerates diabetic wound healing. This provides a new strategy for the clinical treatment of diabetic wounds based on ERβ activation.

### Hyperglycemia-Induced Positive Feed-Forward Loop for ROS Generation

Our results showed that hyperglycemia-induced ROS generation suppresses ERβ expression, and subsequently suppresses its downstream target gene SOD2 (12). The SOD2 suppression then results in additional ROS over-generation. This generates a positive feed-forward loop for ROS generation of ROS, triggering several pathophysiological signaling pathways. This effect remains active after the removal of the original driving force by the switch from hyperglycemia to normoglycemia. This partly explains the potential mechanism of the hyperglycemia memory (8, 9), and our findings show that ERβ expression or activation (28) may be able to break this kind of loop, and subsequently diminish the hyperglycemia memory.

### Hyperglycemia-Induced ERβ Suppression

Our results showed that transient hyperglycemia exposure induces persistent ERβ suppression after switching to normoglycemia. This suggests that ERβ may play an important role in hyperglycemia-induced diabetic complications and delayed diabetic wound healing (29, 30), although the detailed mechanism for hyperglycemia-induced ERβ suppression is still unclear. We have previously showed that SIRT1 regulates ERβ suppression in endothelium and contributes to vascular aging (15). Hyperglycemia-induced decreased SIRT1 activity may potentially suppress ERβ expression (31, 32).

### Potential Effect of ERβ Expression in Diabetic Wound Healing

In this study, we reported that ERβ suppression contributes to hyperglycemia-induced persistent oxidative stress, and subsequently results in delayed diabetic wound healing. Activation of ERβ may break this ROS generation loop and accelerate wound healing. On the other hand, it has also been reported that ERβ regulates the basal expression of ERRα (22), and subsequently regulates nitric oxide (NO) generation via modulation of eNOS (33), together with lipid metabolism and mitochondrial function (14, 15, 34, 35). In this case, the potential effect of ERβ activation on the treatment of diabetic wound healing should be a complicated process. Development of a more specific drug for delayed diabetic wound healing based on ERβ activation with fewer side effects should be in our considerations.

## Conclusions

Taken altogether, our results show that hyperglycemia-induced ROS generation suppresses ERβ expression and subsequently results in SOD2 suppression with further elevated ROS generation. This forms a positive feed-forward loop for ROS generation and delays diabetic wound healing. ERβ agonist DPN, or SOD mimetic MnTBAP, breaks this ROS generation loop, ameliorates the oxidative stress-mediated pathological responses, and subsequently accelerates diabetic wound healing. This provides a potential targeting strategy for diabetic wound healing based on ERβ activation.

## Ethics Statement

The animal protocol conformed to US NIH guidelines (Guide for the Care and Use of Laboratory Animals, No. 85–23, revised 1996), and was reviewed and approved by the Institutional Animal Care and Use Committee from Wuhan University.

## Author Contributions

PY wrote the paper. PY, LL, and WX designed, analyzed the data and interpreted the experiments. LZ and HZ performed vector constructions and gene expression analysis. QR, ZC, ZY, and XH performed statistical analysis and part of the rats experiments. XZ, ML, and MX performed the remaining experiments. All authors read and approved the final manuscript.

### Conflict of Interest Statement

The authors declare that the research was conducted in the absence of any commercial or financial relationships that could be construed as a potential conflict of interest.

## Abbreviations

ChIP: chromatin Immunoprecipitation
DFU: Diabetic foot ulcer
DPN: 2,3-bis(4-Hydroxyphenyl)-propionitrile
ERβ: estrogen receptor β
HIF1α: hypoxia-inducible factor α
HUVEC: human umbilical vein endothelial cells
MnTBAP: Mn(III) 5,10,15,20-tetrakis(4-carboxylatophenyl) porphyrin
${\text{O}}_{2}^{.}-$: superoxide anions
OVX: ovariectomised
PHTPP: 2-Phenyl-3-(4-hydroxyphenyl)-5,7-bis(trifluoromethyl)-pyrazolo[1,5-a]pyrimidine, 4-[2-Phenyl-5,7-bis(trifluoromethyl)pyrazolo[15-a]-pyrimidin-3-yl]phenol
PPT: 4,4′,4″-(4-Propyl-[1H]-pyrazole-1,35-triyl
qPCR: quantitative real-time PCR
ROS: reactive oxygen species
SOD2: mitochondrial superoxide dismutase
STZ: streptozotocin
VEGF: vascular endothelial growth factor.
