# Supplementary material for: ERβ Accelerates Diabetic Wound Healing by Ameliorating Hyperglycemia-Induced Persistent Oxidative Stress
Source: Front Endocrinol (Lausanne). 2019 Jul 24;10:499. doi: 10.3389/fendo.2019.00499 (PMC6667639; doi:10.3389/fendo.2019.00499)
Supplement: Supplementary file 1 [file Data_Sheet_1.docx]

**ERβ Accelerates Diabetic Wound Healing by Ameliorating Hyperglycemia-Induced Persistent Oxidative Stress**

Xueqing Zhou^1,2,^*, Min Li^2,^*, Meifang Xiao^3,^*, Qiongfang Ruan^2^, Zhigang Chu^2^, Ziqing Ye^2^,

Liyan Zhong^3^, Alyssa Chau^2^, Haimou Zhang^4^, Xiaodong Huang^2^,

Weiguo Xie^2,#^, Ling Li^3,#^, Paul Yao^2,3,#^

**Supplemental Information**

**Table S1. Sequences of primers for the real time quantitative PCR (qPCR)**

| Gene | Species | Analysis | Forward primer (5'→3') | Reverse primer (5'→3') |
| --- | --- | --- | --- | --- |
| β-actin | Human | mRNA | gatgcagaaggagatcactgc | atactcctgcttgctgatcca |
| ERα | Human | mRNA | gggaagctactgtttgctcct | ttgaggcacacaaactcctct |
| ERβ | Human | mRNA | atgatgatgtccctgaccaag | acatcagccccatcattaaca |
| HIF1α | Human | mRNA | tttgctggccccagccgct | tctgtaatttttcgttggg |
| SOD2 | Human | mRNA | gcctacgtgaacaacctgaac | tgaggtttgtccagaaaatgc |
| VEGF | Human | mRNA | gccagcacataggagagatga | catttacacgtctgcggatct |
| SOD2 | Human | ChIP | cagcgcaaccaaaactcag | ctgtctgccgtacttgagtgg |
| VEGF | Human | ChIP | gggagccagagaccagtg | cccaaaacttttcccaaactc |
| β-actin | Rat | mRNA | ttccttcctgggtatggaatc | cttctgcatcctgtcagcaat |
| ERβ | Rat | mRNA | tcagcatgaagtgcaaaaatg | ggttctgggagctctctttgt |
| SOD2 | Rat | mRNA | caactcaggttgctcttcagc | ctcaaaagacccaaagtcacg |
| VEGF | Rat | mRNA | tatcttcaagccgtcctgtgt | ctctcctatgtgctggctttg |

FIGURE S1.

**Figure S1. Hyperglycemia-induced persistent ROS generation cannot be diminished by ERα activation.** (a) HUVECs were incubated in hyperglycemia (25mM glucose) for 4 days, and then switched to normoglycemia (5mM glucose) for an additional 4 days, or the cells were treated by 100μM ERα agonist (PPT) on day 4 for subsequent 4 days, and the cells in each day were harvested for the analysis of ROS formation, n=4. *, *P*<0.05, vs day 0 group; †, *P*<0.05, vs day 3 group; #, *P*<0.05, vs day 4 group. Results are expressed as mean ± SEM.

FIGURE S2

**Figure S2. Hyperglycemia-induced persistent SOD2 suppression cannot be restored by ERα activation.** HUVECs were incubated in hyperglycemia (25mM glucose) for 4 days, and then switched to normoglycemia (5mM glucose) for an additional 4 days, or the cells were treated by 100μM ERα agonist (PPT) on day 4 for subsequent 4 days, and the cells in each day were harvested for the analysis of mRNA levels. (a) ERβ mRNA level, n=4. (b) SOD2 mRNA level, n=4. *, *P*<0.05, vs day 0 group; †, *P*<0.05, vs day 3 group. Data are expressed as mean ± SEM.
